# Evaluation of Disulfide Bond Position to Enhance the Thermal Stability of a Highly Stable Single Domain Antibody

**DOI:** 10.1371/journal.pone.0115405

**Published:** 2014-12-19

**Authors:** Dan Zabetakis, Mark A. Olson, George P. Anderson, Patricia M. Legler, Ellen R. Goldman

**Affiliations:** 1 Center for Bio/Molecular Science and Engineering, US Naval Research Laboratory, Washington, DC, United States of America; 2 Department of Cell Biology and Biochemistry, USAMRIID, Frederick, Maryland 21702, United States of America; Russian Academy of Sciences, Institute for Biological Instrumentation, Russian Federation

## Abstract

Single domain antibodies are the small recombinant variable domains derived from camelid heavy-chain-only antibodies. They are renowned for their stability, in large part due to their ability to refold following thermal or chemical denaturation. In addition to refolding after heat denaturation, A3, a high affinity anti-Staphylococcal Enterotoxin B single domain antibody, possesses a melting temperature of ∼84°C, among the highest reported for a single domain antibody. In this work we utilized the recently described crystal structure of A3 to select locations for the insertion of a second disulfide bond and evaluated the impact that the addition of this second bond had on the melting temperature. Four double-disulfide versions of A3 were constructed and each was found to improve the melting temperature relative to the native structure without reducing affinity. Placement of the disulfide bond at a previously published position between framework regions 2 and 3 yielded the largest improvement (>6°C), suggesting this location is optimal, and seemingly provides a universal route to raise the melting temperature of single domain antibodies. This study further demonstrates that even single domain antibodies with extremely high melting points can be further stabilized by addition of disulfide bonds.

## Introduction

Two of the principal measurable parameters relating to protein stability are the melting temperature and the ability to refold into the native state upon cooling. Single domain antibodies (sdAbs) derived from the heavy-chain-only antibodies of camelids and sharks can be characterized in these terms [1]–[4]. Since highly stable proteins are desirable for applications ranging from therapeutics and vaccines to diagnostic reagents, considerable effort has gone into discovering or developing methods of stabilization. In particular, much effort has been invested towards improving the stability of recombinantly-expressed antibody fragments [5]–[10].

The sdAb structure consists of three Complementarity Determining Regions (CDRs) which are highly variable and four framework regions which are highly conserved [11]–[14]. Almost all wild type sdAbs contain one disulfide bond that joins frameworks 1 and 3. This bond spans the interior of the protein and links together two banks of bonded beta-sheets. The removal of this disulfide bond by site-directed mutagenesis results in a significant decrease in melting point and can prevent refolding [8], [15], [16].

The addition of further intramolecular disulfide bonds which form covalent linkages between protein strands has been exploited to improve stability of recombinant antibodies, including sdAbs [17]. Hagihara and coauthors [15] added a novel disulfide bond by using cysteines to replace the native alanine and isoleucine at positions 49 and 70 of a sdAb. These residues are highly conserved in camelid antibodies and span the hydrophobic interior between beta-sheets. The authors achieved a 10°C increase in melting point. Hussack and coworkers [18] studied a group of 6 antibodies into which they added a disulfide bond analogous to Hagihara et al. The melting temperature was improved in all cases (the range was 4–12°C improvement).

Saerens and coworkers [16] studied the effects of having up to three disulfide bonds in one sdAb. The three bonds consist of the bond found in the wild type sdAb, a bond analogous to that of Hagihara et al., and a novel bond connecting CDR1 and framework 3. Among three antibodies tested, stability improvements of up to 19°C were reported. This group also described a sdAb with a naturally-occurring second disulfide linking CDRs 1 and 3 [19] and proposed that in addition to stabilization an extra disulfide bond also rigidifies an antibody and that this can be beneficial for binding affinity.

As previously reported, sdAb A3 is highly thermally stable with a melting point of ∼84°C. It was derived from an immunized llama by selection from a phage-display library and is specific for the Staphylococcal enterotoxin B (SEB) [20], [21]. It contains the conserved disulfide bond between C22 and C99. Previous work has shown that CDR2 plays a critical role in both the affinity and the high thermal stability of sdAb A3 [22]. Structural and mutational studies have been employed to both understand the high melting temperature of sdAb A3 and to engineer additional stability into the protein [8], [23], [24]. In this work we used modeling to predict suitable locations for a number of additional cysteines, designed to form disulfide bonds which would constrain regions involved in the early stages of unfolding. These mutants were then constructed and evaluated for their impact on melting temperature, refolding, and affinity.

## Materials and Methods

### Antibodies and Mutations

The protein sequences of sdAbs A3 and A3-ds were previously published [8], [22]. The crystal structure is from PDB 4TYU [24]. All mutations were introduced by the QuikChange II site-directed mutagenesis kit (Agilent). Sequence verification was completed (MWG Operon) and protein expression was carried out using the pET22b+ vector and Rosetta(DE3) *E. coli* (EMD Millipore). Expression was as previously described [8], [23]. Cultures of 500 ml Terrific Broth were induced with 0.5 mM IPTG. Proteins were recovered from the periplasm by osmotic shock and purified by nickel-affinity chromatography using Ni Sepharose High Performance (GE Healthcare) followed by gel-exclusion liquid chromatography. The final buffer was phosphate-buffered saline, pH 7.4 (PBS). Protein concentration was determined by optical density at 280 nm.

### Protein modelling and mutation selection criteria

Structural regions of sdAb A3 for introduction of disulfide bonds were identified from self-guided Langevin dynamics (SGLD) simulations of the B-chain conformer taken from the crystallographic homodimeric assembly of sdAb A3 [24]. Temperature-based replica exchange was applied to sample conformational space and locate regions that drive the onset of early-stage unfolding of the B-chain. Positional fluctuations were monitored during the simulation trajectory as well as the disruption of native contacts of the sdAb A3 fold topology. Selected regions that undergo early thermal unraveling were analyzed by computing a Cα-Cα coordinate distance matrix and looking for residue side chains and their torsional angles that allow for the potential formation of a disulfide bond in the native conformation.

The overall simulation protocol is similar to our earlier reported protein folding-unfolding modeling studies [25]–[28]. The force field consisted of the CHARMM22/CMAP potential with solvent modeled by the generalized Born molecular volume solvent model. Nonbonded interaction cutoff parameters for electrostatics and van der Waals terms were set at a radius of 22 Å with a 2-Å potential switching function. The SGLD and replica-exchange methods applied are those implemented in the Multiscale Modeling Tools for Structural Biology [29]. A total of 32 replicas were used and exponentially spaced from a temperature of 300 K to 475 K. A simulation time length of 10 ns was applied using an integration time step of 2 fs and the frequency of replica exchanges was set to every 1 ps of simulation.

A final list of A3 disulfide bond candidate substitutions for experimental determination consisted of A50C/A108C, V33C/M105C and Q3C/Y118C. In addition to these identified sites, the A49C/I73C site was modeled from reported studies of introducing a disulfide bond into homologous heavy-chain antibody variable domains [18], [30].

### Circular Dichroism

Protein melting temperatures were determined as previously described [22]. Proteins were diluted or dialyzed into deionized water to a final concentration of about 12 µg/ml. Circular dichroism (CD) was monitored at a single wavelength between 200 and 205 nm as the temperature was raised from 25°C to 95°C at a rate of 2.5°C/min using a Jasco 815 Spectropolarimeter fitted with a Peltier temperature control unit. Data was fit to a sigmoidal curve using IGOR Pro 6 and the melting point is taken to be the inflection point of the curve. Both heating and cooling experiments were conducted to assess the ability to refold into the native state. For each mutant at least two independent protein preparations were measured.

### Fluorescent Dye Assay

Melting temperatures were also determined by binding of a fluorescent dye during protein unfolding as described previously [31]. SYPRO Orange Protein Gel Stain (Sigma) was diluted 1000-fold in a final volume of 20 µl PBS. Final protein concentration was 500 µg/ml. Experiments were carried out with a StepOne Real-Time PCR system (Applied Biosystems). Samples were heated from 25°C to 99°C at a rate of 1% (∼2°C/min) with continuous monitoring of fluorescence using the ROX channel. First derivative of the data was calculated by the manufacturer's software and the melting temperature was taken to be the peak of the derivative data. All measurements were in triplicate.

### Differential Scanning Calorimetry

For Construct 1 the melting point was also measured using a TA Instruments NanoDSC [8]. Protein at a concentration of about 1.6 mg/ml in PBS was degassed and placed into the chamber with a suitable reference buffer. Temperature was raised from 25°C to 120°C at a rate of 1°C/min. Data was collected as µW.

### Surface Plasmon Resonance

Affinity of wild type and mutant antibodies was determined using a ProteOn XPR36 Protein Interaction Array System (Bio-Rad) as described previously [21]. All tests were performed on a GLC sensor chip, which utilizes a general amine coupling and has a compact polymer layer with a binding capacity of approximately one protein monolayer. Following activation of the sensor chip with 0.1 M 1-ethyl-3-[3-dimethylaminopropyl]carbodiimide hydrochloride and 0.25 M N-hydroxysulfosuccinimide, immobilization was performed in 10 mM acetate buffer pH 5.0 for immobilization of SEB (Toxin technologies) at 20 µg/mL on all six lanes. The binding kinetics of each sdAb was determined by rotating the chip and flowing various concentrations (300, 100, 33, 11, 3.7, 0 nM) over the chip at 100 µL/minute in the orthogonal direction for 90 s over the SEB-coated chip and then monitoring dissociation for 600 s. The surface was regenerated at 50 µL/minute with 1% phosphoric acid for 36 s. Data was corrected by subtraction of the zero antigen concentration column as well as by interspot correction.

## Results

### Favorable locations for additional disulfide bonds are present in the sdAb A3 crystal structure

**Figure 1:**
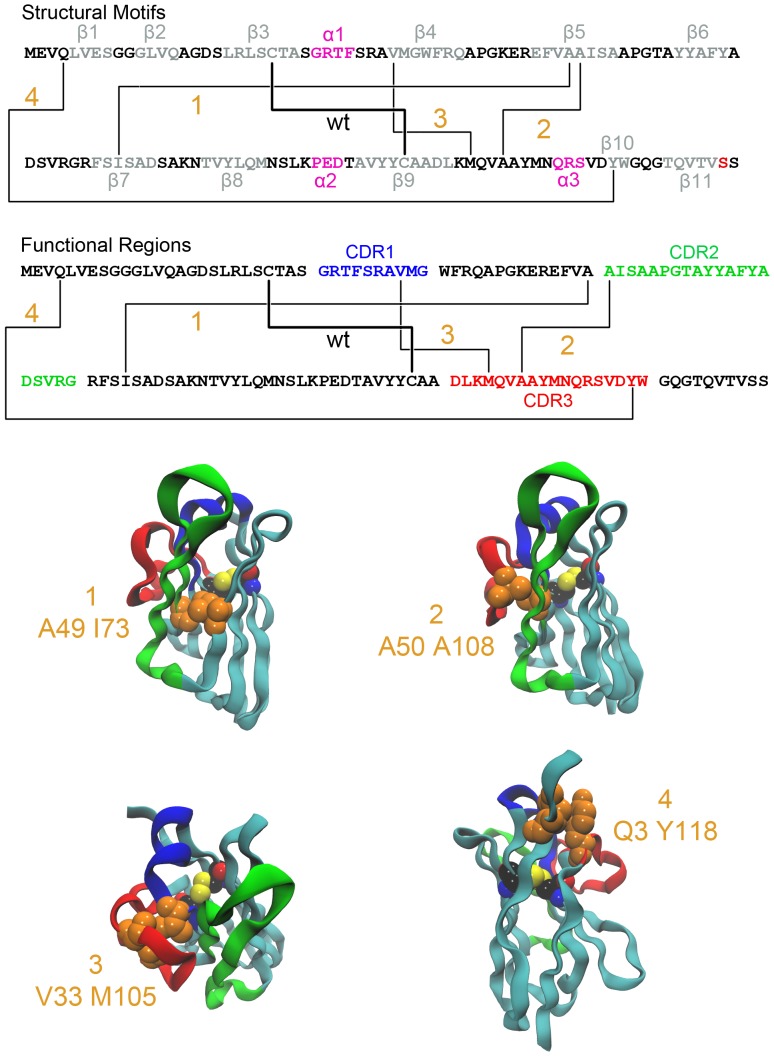
Locations of the disulfide bonds added to sdAb A3 in Constructs 1 through 4. The amino acid sequence of sdAb A3 is given with lines connecting the residues which are changed to cysteine in the constructs as shown. The three dimensional location of each new disulfide bond is shown as an orange-colored space-filling representation of the amino acid pair in the crystal structure of sdAb A3. The CDRs are color coded blue, green, and red, respectively. The native disulfide bond is shown as a standard color space-filling representation.

Positions for inserting second disulfide bonds were proposed within the A3 structural assembly; each set of sites links regions that permit side-chain configurations to satisfy the geometric and distance constraints of bond formation. Fig. 1 shows the locations of the added disulfide bonds within the A3 amino acid sequence and where the cysteine substitutions are positioned within the structure of A3. The A49C-I73C mutations link the two β-sheets of the β-sandwich fold topology by linking β-strands β5 and β7. While the amino acid types lack absolute invariance in the sequence space of known sdAbs, the overall secondary structures are highly conserved within the scaffold framework of the protein fold. The replica-exchange simulations show at high temperatures the two β-sheet regions to exhibit shear dislocations and it was thought that to reduce thermal unravelling the local contact interactions could be strengthened by the introduction of a disulfide bond.

Depending on the method used to assign the secondary-structure of A3, the other proposed sites have at least one residue positioned in an annotated coil or unstructured region. Incorporation of cystines at the site A50-A108 links the β-strand β5 and the approximately 20-residue variable loop region of CDR3. This S-S bond placement was selected to tether the CDR3 during high-temperature excursions, and from the simulations, the region was found to be hypermobile with large collective amplitude movements. From the ensemble of structures generated by the simulations, damping the CDR3 mobility by S-S tethering would conceivably provide a protein folding nucleation site for enhanced configurational stability.

The V33-M105 linkage is the tethering of the CDR1 structural region by reducing flexibility of the N-terminal region of the β4-strand and the CDR3 long-coil segment. From the A3 crystal structure, residues V33 and M105 have side-chain positions showing minimal solvent exposure and are unlikely to be recognized by the binding antigen. Similar to A50-A108, the simulations suggest securing the two segments should help diminish early-stage thermal unravelling and restrict the high mobility of the backbone.

The Q3-Y118 region produced early-onset dislodgement during the simulation trajectory at high temperatures where the intramolecular contacts were observed to be energetically weak. The residue Q3 is located in the N-terminal coil region that precedes β1 and β2, both of which form an extended segment that contacts primarily the first β-sheet of the protein fold. The strands β1 and β2 are of short residue length and appear transient in conformational sampling from the simulations.

### Each double disulfide bond construct has a positive effect on protein stability

The mutations shown in Table 1 were created from the sdAb A3 sequence by site-directed mutagenesis followed by DNA sequence verification. Bacterial periplasmic protein expression was carried out using the pET22b+ vector and Rosetta (DE3) *E. coli*. Protein yield for the double disulfide bond mutants was between 1 and 4 mg per liter of culture.

**Table 1:** Melting temperatures and affinities.

Construct	C22/C99?^a^	Mutations	Location	T_m_ (°C)	Refold	K_D_ (M)
A3	yes	-	-	84	72%	2.3×10^−10^
A3-ds	no	-	-	57	-	0.8×10^−10^
1	yes	A49C I73C	Fr2-Fr3	>90	88%	6.6×10^−10^
2	yes	A50C A108C	CDR2-CDR3	89	83%	0.6×10^−10^
3	yes	V33C M105C	CDR1-CDR3	89	81%	2.1×10^−10^
4	yes	Q3C Y118C	Fr1-CDR3	88	77%	0.6×10^−10^

a Wild type sdAb A3 contains a disulfide bond between cysteines at positions 22 and 99 of the sdAb. Constructs with cysteines at those positions (C22/C99) are listed as "yes".

**Figure 2:**
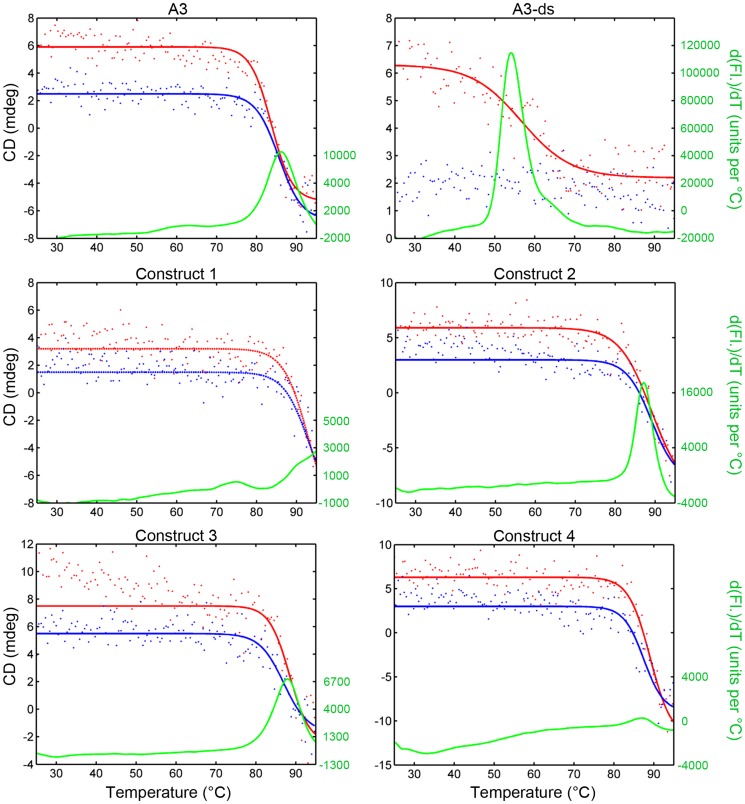
The thermal stability of sdAb A3 and constructs. CD and a fluorescent dye method were used to determine the melting point of the antibodies. CD is represented by red (heating) and blue (cooling) points. The solid lines are a sigmoidal curve fit. See text for an explanation of the dotted lines for Construct 1. The green line is the first derivative of the fluorescence in the dye method. The melting temperature is taken to be the inflection point in the CD heating curve. The dye method peak is in close agreement in most cases.

Proteins were diluted or dialyzed into deionized water to a concentration of 12 µg/ml for determination of melting point by CD. Thermal denaturation curves are shown in Fig. 2 and melting temperatures are reported in Table 1. In all cases the melting point was improved by the addition of an extra disulfide bond. Constructs 2 and 3 had a 5°C improvement when compared with A3. Construct 1 showed the largest increase of at least 6°C. The addition of a second disulfide bond was also observed to increase the apparent refolding percentage for each of the constructs. For comparison we also include results from A3-ds, a variant which lacks the disulfide bond as C22 and C99 have been mutated to alanine and valine respectively; this construct was previously shown to have a lower melting temperature and to have lost its ability to refold after heat denaturation [8], [23].

The CD data for Construct 1 failed to demonstrate a clear inflection point within the maximum range of the instrument (<95°C). Fitting a sigmoidal curve with commercial software yielded an implausible value of 109°C. Subjectively, the curve fit did not appear ideal, particularly in the region of the 90–95°C. Manually fixing the melting point to a lower value gave more satisfying curves. The curve shown (in Fig. 2) is fixed at 93°C and is displayed as a dotted line to represent this fact.

For confirmation each protein was also tested for melting behavior by a fluorescent dye method. Proteins were heated at a concentration of 500 µg/ml in the presence of the dye SYPRO Orange in PBS. The first derivative of the fluorescence typically shows a sharp peak at the melting point of the protein, representing the point at which hydrophobic internal residues become accessible to the dye and thereby enhance the fluorescence.

In most cases the dye result was in good agreement with the CD result. Construct 4 exhibited a low magnitude for the first derivative peak, but was nevertheless consistent with the melting temperature obtained by CD.

**Figure 3:**
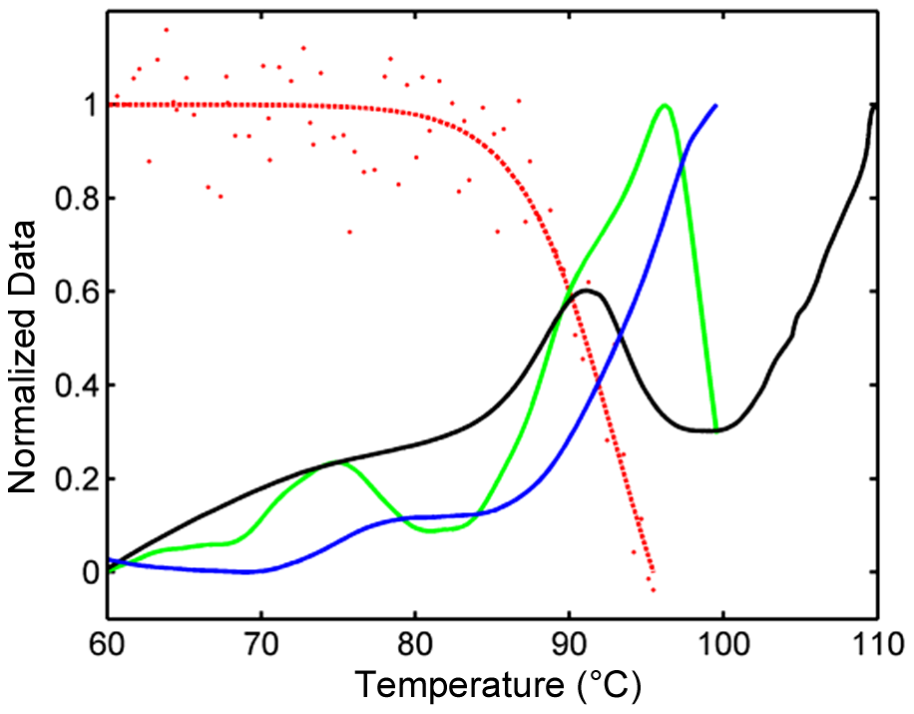
Thermal denaturation of Construct 1 monitored by CD, DSC, and fluorescent dye assay. The high melting point of Construct 1 is ambiguous but above 90°C by multiple techniques. The CD data from Fig. 2 is plotted (red) along with the raw fluorescent dye data (blue), the first derivative of the dye data (green) and the results of differential scanning calorimetry (black). All data is normalized from zero to one in the range of 60–110°C for best visibility of the changes.

As with the CD data, the fluorescent dye results were ambiguous for Construct 1 as well. The results of the various melting point determinations for Construct 1 are shown in Fig. 3 where the red data represents the CD heating results. In this figure, all data for temperatures of 60–110°C is normalized to the range of 0–1. Both the raw fluorescence (blue) and first derivative (green) are shown. A slight shoulder is observed at about 90°C. There is also a broad peak at about 75°C which does not correspond to any observable event by CD. The main observable peak at about 96°C is aberrantly shaped and may be artifactual. Deflections in the fluorescent data are often observed with this instrument within the last few degrees of the maximum temperature (99°C).

The melting point for Construct 1 was also determined by differential scanning calorimetry (DSC). The normalized data is shown in black in Fig. 3 and displays a melting temperature of 91°C. Since the results for different techniques diverge for Construct 1, we can only claim that the melting point is greater than 90°C.

### Disulfide bonds have little effect on sdAb A3 affinity

**Figure 4:**
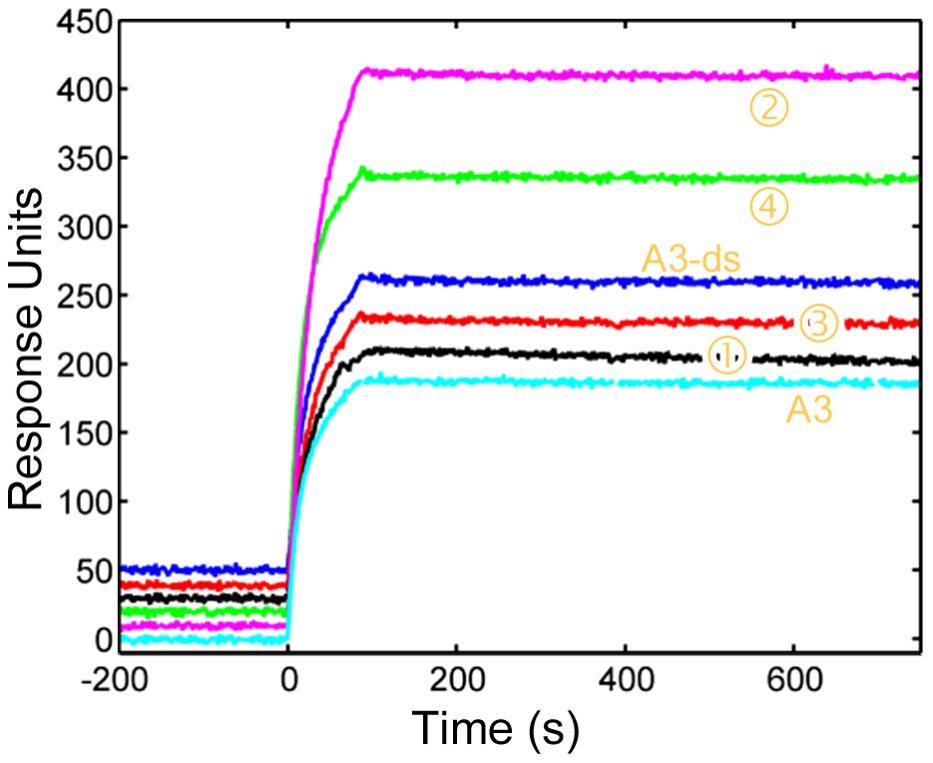
Binding affinity of mutant constructs. Surface plasmon resonance data is shown for each sdAb. Protein concentration was 300 nM and the data are offset by 10 units each for clarity of the base line. Construct identity is shown by orange labels. Little to no effect on the on-rate or off-rate is observed.

In order to determine the functional activity of these constructs experiments were carried out by surface plasmon resonance [8], [21]. The results, reported in Table 1 and Fig. 4, show that there was limited effect of any of these mutations and the dissociation constant (K_D_) varied by less than a factor of four from the wild type value. As can be seen in Fig. 4, all the variants have excellent affinity, showing similar slow off rates. Signal differences were observed due to loss in binding activity due to denaturation of the SEB on regeneration of the chip that occurred during the course of the measurements, but did not impact the calculated binding constants. As observed previously, removing the disulfide bond resulted in a dramatic decrease in melting temperature; however absence of the disulfide bond did not adversely change affinity [8]. Prior work has shown that CDR2 is the main determinant of affinity for sdAb A3 [22]. Only Construct 2 involves CDR2 and then only at the periphery. Thus, it is not surprising that these mutations have no detrimental effects.

### An attempt to produce a sdAb with 5 disulfide bonds failed

Given the success of each additional disulfide bond in improving the protein stability it was decided to make and test a construct featuring all five bonds in one protein. Unfortunately, expression of this construct failed to yield significant protein after multiple attempts. We suspect that the protein is unstable due to malformation of aberrant disulfide bonds among the ten cysteines which may prevent the attainment of an even approximately correct tertiary structure.

## Discussion

In this paper we demonstrated that the addition of a second disulfide bond to a thermally-stable sdAb successfully increased the melting temperature in each of four cases. The ability to refold after denaturation was also improved in each case.

One of our examples, Construct 1, is analogous to the alanine-isoleucine (A49C/I70C) substitution described by Hagihara et al [15]. Since these are highly conserved residues, and multiple examples have been published, this appears to be a general method for stabilization of sdAbs.

Natural antibodies with disulfide bonds in addition to the wild type are known. Often, the variable region from a camel heavy-chain-only antibody (V_HH_) contains an extra non-canonical cysteine within CDR1 (position 31, 33, or 34) which pairs with a cysteine in CDR3 to form an inter-loop disulfide bond [11], [32]. Additionally, non-canonical disulfide bonds have been reported in camel V_HH_ between framework 2 and CDR3 and between CDR2 and CDR3 [32]. In the alpaca there are also reports of V_HH_ examples with disulfide bonds connecting the start of CDR2 with CDR3 [33], [36], [37].

Although a putative additional disulfide bond has been observed in two llama V_HH_ subfamilies, in most cases the llama V_HH_ lacks such a bond [34], [35]. The vast majority of llama-derived sdAbs reported in the literature contain only the disulfide bond in the conserved position. Nonetheless, llama-derived sdAbs show the same refolding ability as dromedary antibodies and are able to bind their cognate antigens with high specificity and affinity [38], [39].

Two of our llama constructs are similar to those found naturally in camels (Constructs 2 and 3) [11], [32] and alpacas (Construct 2) [33], [36], [37].

In all cases, our choice of mutants was based on the hypothesis that a disulfide bond in the respective location would prevent an early unfolding step during protein melting. This, we surmised, would be an effective means to increase overall stability, and has been shown to be successful in stabilizing a ricin immunogen [27]. Constructs 2 and 3 are similar in that they tie down the long CDR3 loop which is shown by the crystal structure to be folded back over the vestigial V_H_–V_L_ interface. Construct 1 is most similar in nature to the disulfide bond found in wild type proteins in that it spans the hydrophobic core of the protein and links the two sets of beta-sheets which comprise the basic protein structure. Construct 4 is very much on the exterior of the protein, but ties together the amino- and carboxy-termini (position 3 linked to position 118, which is 11 amino acids from the end). This bond also links the beta-sheet sets, and on the end at which we might naturally expect them to separate.

All of the mutations resulted in an improved melting temperature and also an improved ability to refold after thermal denaturation. As the results were not radically different between mutants, it is impossible to draw firm conclusions about the relative value of the underlying hypotheses. Construct 1 worked the best, possibly because it crosses the internal core of the protein in a fashion similar to the disulfide bond found in wild type antibodies. This may make Construct 1 more effective on its own as compared to bridges created at more peripheral sites. Alternately, Construct 1 may have superior additive effects as a complement to the C22/C99 disulfide bond. As a pair these bonds may function to stabilize the central structure to a degree greater than the sum of their individual contributions.

The improvement in refolding after thermal denaturation may be due to one or both of two causes. First, since the CD experiment is limited to 95°C, the higher melting proteins may not have been fully and continuously unfolded. In particular, Construct 1 will have been in pseudo-equilibrium between folded and unfolded states even at the highest temperature. This may reduce the tendency to aggregate by association of hydrophobic regions. Alternatively, the additional disulfide bond will reduce the flexibility and freedom of conformation, even for the unfolded state. This may also reduce the ability of hydrophobic regions from forming undesirable associations. As long as additional disulfide bridges do not introduce harmful steric properties into a protein it could be expected that they will assist in the refolding of a thermally denatured protein.

None of the novel mutations created serious disruption to the binding affinity. This is a useful result since 3 of the four tested mutations involved one or more CDR. As long as the disulfide bonds are placed in sterically appropriate locations, it seems likely that the wild type structure will be preserved without damage to the function of the protein. It is important to verify that binding properties are unchanged with the addition of exogenous disulfide bonds as decreased affinity has been reported in some cases [16], [40].

We have shown that even a sdAb with a very high melting temperature can benefit from additional disulfide bonds. When designed based on an understanding of the protein structure it appears there is a high probability of success. The disulfide bond described by Hagihara et al. (our Construct 1) uses conserved residues and is likely to work in most cases. Likewise, mutations that take an antibody sequence closer to a known natural example from another species, such as to camelize (Construct 3) or to alpacanate (Construct 2), are also credible pathways to success. Our Construct 4 is not similar to any known antibody, but through structural considerations was determined to be a valid option. All of the tested mutants had improved stability without a substantial sacrifice of function.
